# Impaired ATF3 signaling involves SNAP25 in *SOD1* mutant ALS patients

**DOI:** 10.1038/s41598-023-38684-8

**Published:** 2023-07-25

**Authors:** Volkan Yazar, Julia K. Kühlwein, Antje Knehr, Veselin Grozdanov, Arif B. Ekici, Albert C. Ludolph, Karin M. Danzer

**Affiliations:** 1 German Center for Neurodegenerative Diseases (DZNE), 89081 Ulm, Baden-Wüerttemberg Germany; 2 Department of Neurology, University Clinic, University of Ulm, Albert-Einstein-Allee 11, 89081 Ulm, Baden-Wüerttemberg Germany; 3 Institute of Human Genetics, University Clinic Erlangen, Friedrich-Alexander-University Erlangen-Nürnberg, 91054 Erlangen, Bavaria Germany

**Keywords:** Data processing, Amyotrophic lateral sclerosis, Data integration

## Abstract

Epigenetic remodeling is emerging as a critical process for several neurodegenerative diseases, including amyotrophic lateral sclerosis (ALS). Genetics alone fails to explain the etiology of ALS, the investigation of the epigenome might therefore provide novel insights into the molecular mechanisms of the disease. In this study, we interrogated the epigenetic landscape in peripheral blood mononuclear cells (PBMCs) of familial ALS (fALS) patients with either *chromosome 9 open reading frame 72* (*C9orf72*) or *superoxide dismutase 1* (*SOD1*) mutation and aimed to identify key epigenetic footprints of the disease. To this end, we used an integrative approach that combines chromatin immunoprecipitation targeting H3K27me3 (ChIP-Seq) with the matching gene expression data to gain new insights into the likely impact of blood-specific chromatin remodeling on ALS-related molecular mechanisms. We demonstrated that one of the hub molecules that modulates changes in PBMC transcriptome in *SOD1*-mutant ALS patients is ATF3, which has been previously reported in an *SOD1*^G93A^ mouse model. We also identified potential suppression of *SNAP25*, with impaired ATF3 signaling in *SOD1*-mutant ALS blood. Together, our study shed light on the mechanistic underpinnings of *SOD1* mutations in ALS.

## Introduction

Amyotrophic Lateral Sclerosis (ALS) is characterized by a progressive loss of upper and lower motor neurons, leading to progressive weakness and atrophy of limb, bulbar and respiratory muscles^1^. Most cases are sporadic, while approximately 10% display a familial (fALS) involvement^2^. Since genetics alone fails to explain the etiology of ALS, the investigation of the epigenome might provide novel insights into disease mechanisms^3^. The epigenome can be considered as a dynamic template that flexibly adapts to diverse stimuli, and endows cells with an individual gene expression profile, without altering the genetic code^4^. Many different risk factors accumulate throughout life and bequeath epigenomic aberrations in addition to genetic susceptibility^5^. Hence, their involvement might be relevant in late-onset diseases, such as ALS^6^. Epigenetic changes, such as modifications in histone proteins or DNA methylation, have been reported to play a role in the development of ALS.

Epigenetic information is stored as covalent modifications of DNA or its packaging histones. Post-translational modifications on histones tightly regulate the degree of chromatin compaction and accessibility of DNA by adjusting the chromatin structure into relatively accessible or inaccessible subdomains depending on their chemical properties^7–9^. Only highly accessible regions allow for interaction between architectural proteins, regulatory elements, and the transcriptional machinery to the DNA^7^. Hence, they are key regulators of transcription, genomic stability, maintenance of normal cell growth, differentiation, and development^10^. Among all histone modifications, the repressive trimethylation of lysine 27 on histone H3 (H3K27me3) might be of particular interest in the context of ALS for several reasons. First, increased levels of this histone mark were observed in aged animal models^11^ and aging is one of the main risk factors for ALS. Second, the modification and its modifying enzymes were implicated in neurogenesis and neuronal differentiation^12,13^, the pathways known to be dysregulated in ALS. Third, previous studies linked H3K27me3 with loss-of-function toxicity in *C9orf72* ALS/FTD patients^14^, providing further evidence for a likely involvement of H3K27me3 in the disease pathogenesis. All this evidence makes H3K27me3 and its effect on the chromatin structure an interesting target for a genome-wide investigation. In this study, we interrogate the epigenetic landscape in peripheral blood mononuclear cells (PBMCs) of fALS patients with either *chromosome 9 open reading frame* 72 *(C9orf72*) or *superoxide dismutase 1* (*SOD1)* mutation and aimed to identify key epigenetic footprints of the disease. To this end, we used well-established omics techniques, including chromatin immunoprecipitation targeting H3K27me3 (ChIP-seq) and gene expression profiling paired with in-depth bioinformatics analysis to gain new insights into the impact of chromatin remodeling on ALS-related molecular mechanisms.

## Results

### H3K27me3 profile of ALS PBMCs

**Figure 1:**
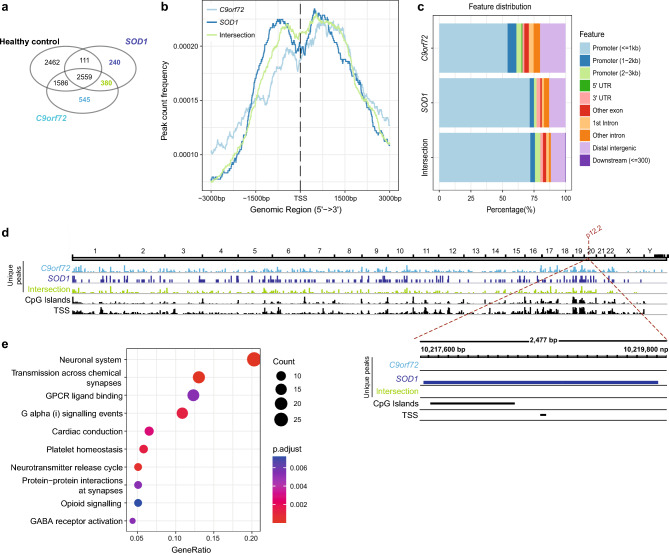
H3K27me3 profile of fALS PBMCs. **(a) A** Venn diagram that shows the number of consensus peaks identified after ChIP-seq targeting H3K27me3 in PBMCs of fALS or healthy controls, with a particular focus on 3 different sectors (unique peaks for *C9orf72* in pale blue, unique for *SOD1* in dark blue, and shared between both ALS groups, also called the ‘intersection’, in green). **(b)** An average profile plot that displays the genomic localization of the consensus peaks in the *C9orf72*- and *SOD1*-specific group, as well as the intersection group, confirming that the peaks overlap with the TSSs of the target genes. **(c)** Distribution of H3K27me3 peaks across genomic functional regions demonstrating highest degree of enrichment for promoter proximal sites (i.e., ± 3 kb around the TSS) in *SOD1*-mutant patients. **(d)** Genome-wide visualization of the consensus peaks per group, showing a high degree of overlap with the genomic elements related to transcription. *SNAP25*, one of the genes with an *SOD1*-specific H3K27me3 peak around the associated TSS, is also given in the figure. GRCh38 was used as reference genome, regions with a high frequency of CG sites are associated with promoter regions of genes in mammalian genomes (CpG islands) and transcription start sites (TSS) of the eukaryotic promoter database. **(e)** The pathway level enrichment by KEGG identifiers of neuronal terms within the peak-associated genes shared between both ALS groups, excluding healthy controls.

To explore whether H3K27me3 is differentially marked in ALS PBMCs, we performed a genome-wide analysis of H3K27me3 occupancy using ChIP-seq in a comparison with 3 sample groups (i.e., healthy control and two groups of ALS samples carrying either *C9orf72* or *SOD1* mutations) (Fig. 1a, Supplementary Table S1). After a successful peak calling (Supplementary Fig. S1-2), we identified unique or shared sets of consensus peaks in both ALS groups (unique peaks for *C9orf72* in pale blue, unique for *SOD1* in dark blue, and shared between both ALS groups in green in Fig. 1a, Supplementary Table S2). The peak count frequencies for these 3 subgroups of peaks revealed an enrichment of read coverage around the transcription start sites (TSSs) of the target genes, as expected (Fig. 1b). The highest degree of peak enrichment was found around the promoter proximal sites for the *SOD1*-specific group of peaks (Fig. 1c). Overall, the highest signal intensities across the genome were also detected for the same group of peaks (Fig. 1d).

Since synaptic proteins, such as synaptotagmin and synaptosomal-associated protein 25 (SNAP-25), were reported to be associated with neurodegenerative, as well as neuropsychiatric diseases^15,16^, we turned our attention to H3K27me3 peaks around the *SNAP25* gene body. We found such a peak around the *SNAP25* TSS solely in the *SOD1* group that overlaps the genomic elements related to transcription (Fig. 1d). Finally, taking the peak-associated genes shared between both ALS groups and unique to ALS as input, the pathway level annotation analysis by KEGG identified an enrichment in neuronal system-related categories (Fig. 1e, Supplementary Fig. S3), suggesting a relevance of a neuronal signature in ALS PBMCs.

### SNAP25 is central to the interaction network composed of the peak-associated genes exclusively found in *SOD1* samples

**Figure 2:**
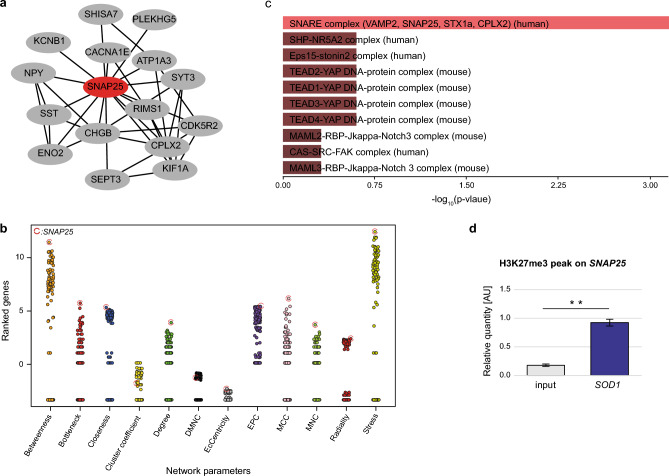
SNAP25 is central to the interaction network composed of the peak-associated genes exclusively found in *SOD1* samples. **(a)** Taking the peak-associated genes unique to *SOD1* group as input, a STRING analysis described *SNAP25* (the red node) as a hub gene in the interaction network. **(b)** Topological analysis of the shown network using cytoHubba revealed *SNAP25* as the central element of the network. Of all 11 network features tested, the “degree” (which correlates well with the essentiality of a gene) also identifies *SNAP25* as the top gene in the network. **(c)** *SNAP25-*related functional terms from the CORUM (protein complexes by LC–MS/MS) database enriched in the query genes appear at the top of the list ranked by significance. Shown are the top hits ordered by their p-value. **(d)** ChIP-H3K27me3 immunoprecipitated DNA was used to verify in silico findings by ChIP-qPCR measuring the H3K27me3 occupancy at the *SNAP25* promoter in the *SOD1* (n = 3) sample group. The input (n = 3) serves as negative control. Bars are shown as mean ± SEM. **p < 0.01, unpaired, parametric t-test.

We then explored the key nodes in the peak-associated genes unique to *SOD1* group using STRING interaction network based on physical and functional associations (Supplementary Fig. S3). The 1st stage nodes of the top-ranking gene (*SNAP25*) by the "degree", which is the most informative network feature in this context and correlates well with the essentiality of a gene, appeared highly interconnected (Fig. 2a). An in-depth network analysis using a set of topological algorithms revealed that *SNAP25* ranked first by 10 of the 12 different network features evaluated in this analysis (Fig. 2b). Taking the peak-associated genes unique to *SOD1* group as input, several functional enrichment analyses found *SNAP25*-related identifiers as the top terms of the list ranked by significance (Fig. 2c, Supplementary Fig. S3). Together with SNAP25, other components of the SNARE complex that help mediate the fusion of vesicles with membrane-bound compartments appeared significantly enriched (see Supplementary Fig. S3 online). Lastly, to validate physical interaction between H3K37me3 and *SNAP25*, we performed ChIP-qPCR and confirmed the H3K27me3 signature at the *SNAP25* promoter region. Overall, these validation results are in line with the in silico findings (Fig. 2d).

### ATF family proteins act on the PBMC transcriptome in ALS caused by *SOD1* mutations

**Figure 3:**
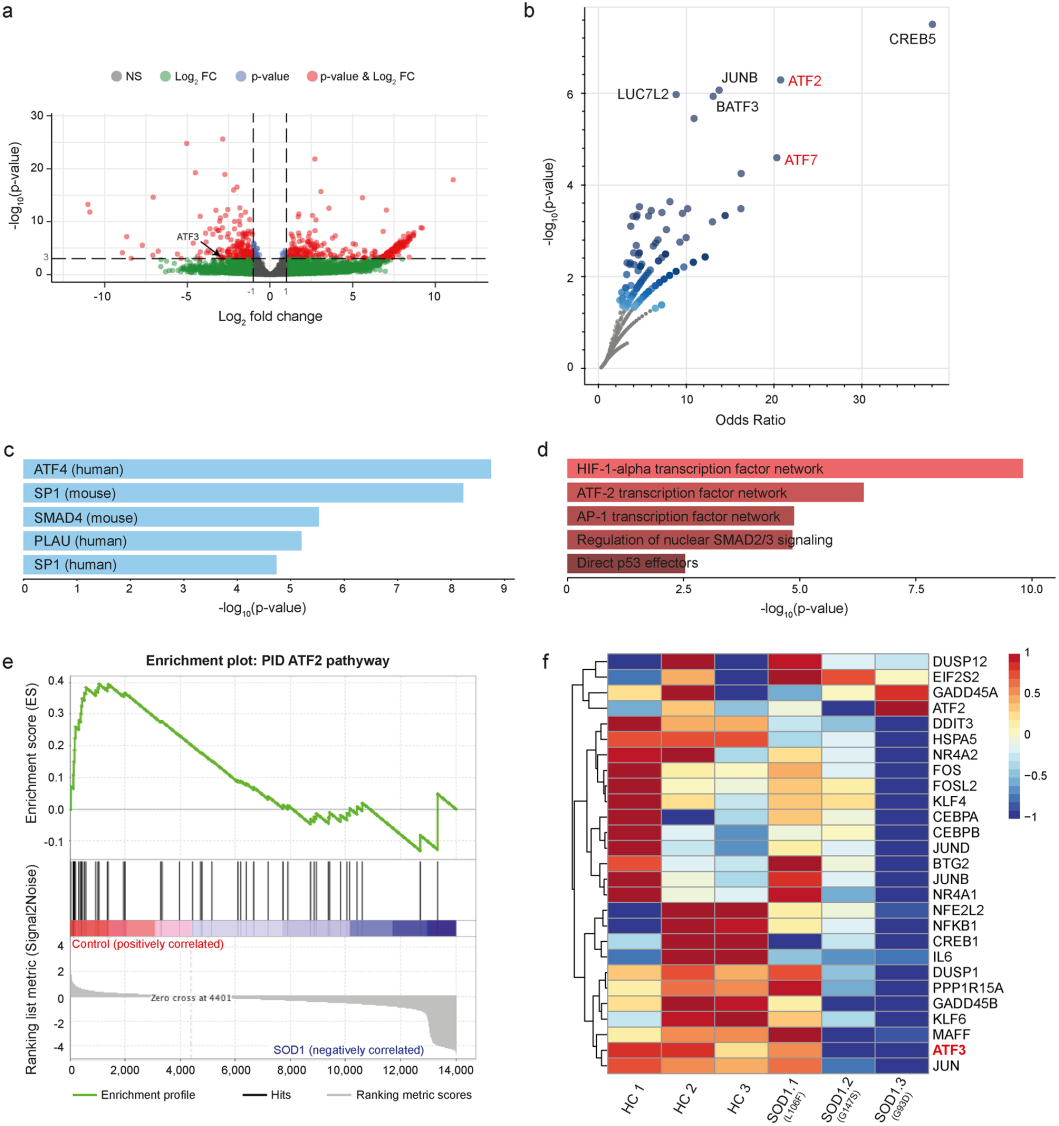
ATF family proteins act on the PBMC transcriptome in ALS patients with *SOD1* mutations. **(a)** A volcano plot with a characteristic pattern showing the significance of each gene as a function of fold change (FC). Each red dot refers to a significant gene at the p-value and the FC cutoff after differential expression analysis of *SOD1*-mutated ALS patients (n = 3) and healthy controls (n = 3). The significance of each gene is shown as a function of fold change. Red dots represent significant genes at the p-value cutoff 1e-03 and the FC cutoff 2.0. **(b)** The functional enrichment analysis using the LC–MS/MS-based BioPlex protein–protein interaction database (2017) revealed ATF family proteins as one of the most associated gene sets with the highest significance in the downregulated DEGs. **(c)** Using the same input genes as in c), a search in the TRANSFAC and JASPAR dBs for TF binding motifs at the corresponding promoter regions identified ATF4 as the most significantly associated TF. **(d)** Of all cell signaling pathways in the NCI-Nature pathway dB (2016), ATF2 TF network appeared as a gene set that is highly enriched in the same input genes used in c). Shown are the top hits ordered by their p-value. **(e)** A gene set enrichment analysis (GSEA) reported ATF2 pathway as highly enriched in *SOD1*-mutant ALS patients, confirming the concerted effect that impaired ATF family TF pathways in ALS PBMCs. **(f)** A heatmap of the ATF3 top 50 interactors (score > 0.400; 1st shell interactors of ATF3) from the STRING dB that indicates *SOD1*.3 with *G93D* mutation contributes mostly to the impaired ATF3 signaling. Input data are publicly available (GSE106443 and GSE115259)^17^.

Next, we asked which direct target genes are associated with H3K27me3 peaks around the promoter region in *SOD1* mutation carriers. In this regard, we used the matching expression data (GSE106443 and GSE115259)^17^ from a public repository of ALS PBMCs with various *SOD1* mutations. Analysis of differential expression between healthy control and *SOD1*-mutant ALS PBMCs revealed 635 significantly down- and 1406 significantly upregulated genes (Fig. 3a, Supplementary Fig. S4). Since the ontology terms enriched in the differentially expressed genes (DEGs) that were upregulated in the ALS group did not converge on a common functional pathway or an interaction network related to neurodegeneration, we did not follow up on upregulated DEGs within the scope of this work. In contrast, for downregulated DEGs in the ALS group functional enrichment analysis using the large scale, LC–MS/MS-based BioPlex protein–protein interaction dB (2017) revealed ATF family proteins as one of the most associated gene sets with the highest significance (Fig. 3b). Moreover, multiple lines of evidence confirmed at the ontology level that ATF family proteins were associated with downregulated DEGs in the ALS group (Fig. 3c–e). More specifically, *ATF-2*, *-3*, *-4*, and *-7* have been found associated with ALS PBMCs carrying a pathogenic *SOD1* mutation in a range of functional annotation databases (Fig. 3b–e). Besides, we conducted a gene set enrichment analysis (GSEA) to identify the gene sets enriched between control and *SOD1*-mutant ALS sample groups. One of the top enriched gene sets was ATF2 signaling pathway (NES: 1.60, nominal *p*-value < 0.001) (Fig. 3e), together with ATF4 signaling (NES: 1.41, nominal *p*-value < 0.05) (Supplementary Fig. S4). In other words, using different in silico approaches we verified a concerted effect that impairs ATF family transcription factor signaling in *SOD1* mutant-ALS PBMCs. We demonstrated using a heatmap that the genes in the leading edge, which include *ATF3*, *ATF4*, and *ATF6*, were also downregulated in the disease group (Supplementary Fig. S4). Lastly, considering the 1st shell interactors of *ATF3* (i.e., the top 50 genes associated with *ATF3* at an interaction score > 0.400 from the STRING dB), we found that an ALS *SOD1*^*G93A*^ mutation carrier contributed mostly to the potentially impaired ATF3 signaling in ALS linked to *SOD1* group (Fig. 3f).

### ATF3 is a key driver of transcriptional regulation in *SOD1*-mutant ALS PBMCs

**Figure 4:**
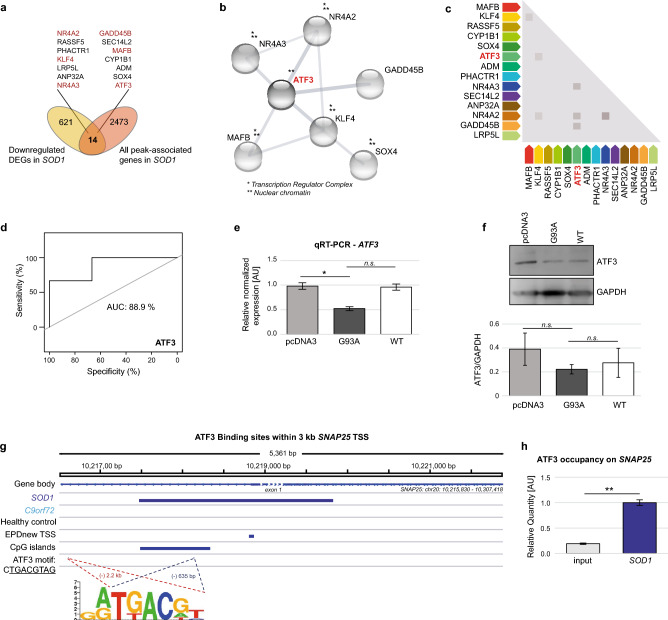
ATF3 is a key driver of transcriptional regulation in *SOD1*-mutant ALS PBMCs. **(a)** By comparing the peak associated genes of the H3K27me3 ChIP-seq from *SOD1*-mutant ALS patients with the RNA-seq data of the differentially downregulated genes (*SOD1* n = 3 (*SOD1L106F, SOD1 G147S, G93D*; HC n = 3), we identified 14 genes that were shared between the two gene sets (with the significance of the overlap by a hypergeometric test: 4.33e-17). These shared genes were found to be orchestrated by **(b)** ATF3 (the enrichment p-value: 5.75e-09) as a part of the sub-network constructed based on physical and functional associations. **(c)** Given the network in b), *ATF3* appeared highly co-expressed with the other elements in this network, implying a likely co-regulation of these genes by ATF3 in the ALS context. **(d)** A machine learning-based approach to evaluate diagnostic ability of a binary classifier system, ROC analysis, showed that ATF3 can distinguish between sample groups. The area under the ROC curve (AUC value) was 0.89 for ATF3 (an AUC value > 0.8 means a good classifier). **(e)** HEK293 cells transfected with either *SOD1*^*G93AL1L2*^ plasmid or pcDNA3 or wild-type SOD^L1L2^ (WT) plasmids validate *ATF3* downregulation using qRT-PCR. Gene expression levels were normalized to the average expression of four housekeeping genes (*B2M*, *Pol2R*, *RNU6*, *TBP).* **(f)** Western Blot analysis of ATF3 expression in HEK lysate after transfection with pcDNA3, *SOD1*^*G93AL1L2*^ and SOD1^L1L2^ (WT) plasmids (loading control: GAPDH). Bars are shown as mean ± SEM. **p < 0.01, unpaired, parametric t-test. **(g)** A view of the UCSC Genome Browser with custom tracks option enabled to show ATF3 binding motifs at the promoter proximal site of *SNAP25*. The only sample group with a H3K27me3 peak overlapping this region is *SOD1*. The set of EPDnew TSS regions was obtained from the Eukaryotic Promoter Database (EPD) for the GRCh38/hg38 genome assembly used throughout the analysis pipeline. The ATF3 binding motif is available online at uniprot.org. **(h)** ChIP-H3K27me3 immunoprecipitated DNA was used for ChIP-qPCR surveying ATF3 occupancy on *SNAP25* promoter in *SOD1* sample group (n = 3). n_input_ = 3. ATF3 physically binds to the *SNAP25* promoter region proximal to the TSS likely to modulate *SNAP25* expression. Bars are shown as mean ± SEM. **p < 0.01, unpaired, parametric t-test.

To investigate transcriptional regulatory mechanisms which might associate ATF3 with *SOD1*-mutant ALS patients any further, we integrated H3K27me3 repressive histone marks identified during ChIP-seq data analysis (specifically for *SOD1*-mutant patients) with significantly suppressed genes detected in the expression data of the public repository (*SOD1* n = 3; HC n = 3)^17^. We identified 14 shared genes with a high significance of the overlap (*p*-value = 4.33e-17) (Fig. 4a). To attain deeper knowledge of the molecular interactions of these 14 query genes based on physical and functional associations, a STRING PPI analysis reported *ATF3* as a hub gene in the network (the enrichment *p*-value: 5.75e-09) (Fig. 4b). Given the network in Fig. 4b, *ATF3* appeared highly co-expressed with the other elements in the network (Fig. 4c), implying a likely co-regulation of these genes by *ATF3* in the ALS context^18^. With regard to the ALS-dependent changes in expression levels, these genes (*ATF3*, *SOX4*, *KLF4*, *MAFB*, *GADD45B*, *NR4A2*, and *NR4A3*) were found to have a varying range of downregulation (FC: 2.6, 2.0, 1.7, 2.1, 3.0, 2.9, and 3.1, respectively) with high statistical significance (FDR-adjusted *p*-values up to 1.5e-06) (Supplementary Fig. S5). As an initial step of our validation efforts, a machine learning-based approach to evaluate diagnostic ability of a binary classifier system, ROC analysis, showed that *ATF3* expression can distinguish between sample groups well (Fig. 4d). Given that the area under a ROC curve (the AUC value) of 0.8 or more implies outstanding classifier for phenotype discrimination in terms of both sensitivity and specificity, the AUC values calculated for *ATF3* (0.89) was highly promising. To confirm this finding in vitro, we used HEK293 cells transfected with *SOD1*^G93AL1L2^ or *SOD1*^*L1L2*^ wild type, as well as controls*.* As expected, we were able to detect a significant decrease in the expression of *ATF3* in *SOD1*^*G93AL1L2*^ transfected cells using RT-qPCR (Fig. 4e). To add further evidence on protein level, we performed Western Blots with the same sample groups. Although not statistically significant a concordant downregulation of ATF3 in the *SOD1* constructs seemed to be present (Fig. 4f).

### ATF3 interacts with *SNAP25* promoter region proximal to the TSS

After observing the likely involvement of ATF3 in *SOD1*-mutant ALS pathogenesis observed in PBMCs and the potential centrality of *SNAP25* within the network of genes localized to transcriptionally silenced H3K27me3-marked chromatin unique to the *SOD1* sample group, we investigated first in a bioinformatics framework then experimentally whether *SNAP25* is among the target genes ATF3 acts on. We found that the promoter-proximal site of *SNAP25*, as shown with the overlapping CpG island and the predicted TSS, contains a binding motif for ATF3 (G(A/G)TGACGT) at two different regions, namely − 2.2 kb and − 635 bp with respect to the TSS (Fig. 4g). This observation implied a direct regulatory effect of ATF3 on *SNAP25* as one of its potential target genes. The only sample group with an H3K27me3 peak overlapping this region was *SOD1*, as expected. Ultimately, we performed experimental validations using ChIP-qPCR to confirm whether ATF3 interacts with the *SNAP25* promoter region. ATF3 was found to physically bind to the *SNAP25* promoter region proximal to the TSS, likely to modulate *SNAP25* expression levels (Fig. 4h). Again, the validation results were in line with the in silico findings presented in this work. Another line of evidence which further corroborates these findings at protein level came from the combined analysis of the current data and a previously published^19^, qualitative LC–MS/MS proteomics data set that was generated from spinal cord and cortex tissues of ALS patients. The functional enrichment results of the common genes obtained from various annotation libraries suggested that alterations in ATF family TFs and *SNAP25* play a role in the ALS pathology (Supplementary Fig. S6).

## Discussion

In this study, we set out to characterize epigenetic signatures combined with subsequent expression changes unique to ALS in human PBMCs, which might ultimately serve as a potential source of biomarkers in future. We achieved a part of this by constructing a genome-scale, detailed map of the H3K27me3 binding regions in PBMCs from healthy and ALS donors. After combining our in-house ChIP-seq data set with the matching expression data from a public repository, we found direct target genes in ALS that are associated with H3K27me3 peaks around the promoter region, particularly in *SOD1*-mutant ALS patients. The principle finding of this work is the observation that one of the hub molecules that modulates changes in PBMC transcriptome in *SOD1*-mutant ALS cases is ATF3. Accompanying the regulatory role of ATF3 in this molecular network was the deregulation of a set of genes involved in cellular stress, inflammation, and several neurological phenotypes.

Multiple lines of evidence from an ALS transgenic (*SOD1*^*G93A*^) mouse model showed that forced expression of *ATF3* in motor neurons improved motor functions by promoting motor neuron survival and retaining muscle innervation, as well as improved motor performance^20^. Of note, in our *SOD1* sample group, it was a patient carrying the *SOD1*^*G93D*^ mutation that was found to contribute mostly to the impaired ATF3 signaling in ALS. Together, human blood and mouse brain samples with a *SOD1* mutation in the same position seem to converge on the same molecular dysfunction (i.e., impaired ATF3 signaling) within the context of ALS.

Encoding a member of the activating transcription factor (ATF) family proteins and belonging to the ATF/cyclic AMP responsive element binding family of proteins, *ATF3* gene is induced by various stress signals and serves as a hub of the cellular-response network^21^. More specifically, ATF3 is widely known in mammalians to modulate inflammatory response, which is intimately related to the pathophysiology of a wide spectrum of diseases, including neurodegeneration and ALS. While activating a set of target genes depending on the stress signal, ATF3 might as well repress the expression of another set of genes in different cell types, including neurons and glia. Though the basal levels of *ATF3* expression in neurons and glia are relatively low, an immense boost of expression is generally observed upon stress signal, which in turn leads to neuronal survival and regeneration of their axons^22,23^. Taken together, identifying *ATF3* as the hub molecule in the downregulated DEGs with a repressive histone mark around the TSS in *SOD1*-mutant ALS PBMCs in the course of this work appears as a good starting point to elaborate on this potential link between ATF3 and neurodegeneration in ALS brain with the same genotype.

Since we also identified *SNAP25* as the core element of the molecular network composed of H3K27me3 peak-associated genes exclusively found in the *SOD1*-mutant patients, we asked whether the *SNAP25* TSS might harbour a ATF3 binding site. Indeed, a ATF3 motif could be identified within 3 kb of *SNAP25* TSS and ATF3 occupancy on *SNAP25* could be validated using ChIP-qPCR. *SNAP25* encodes a plasma membrane protein which, along with syntaxin and the synaptic vesicle protein VAMP/synaptobrevin, constitutes the SNARE (soluble N-ethylmaleimide-sensitive factor attachment protein receptor) docking complex for many intercellular signaling processes^16^. SNAP25 regulates a set of voltage-gated calcium channels as well, thereby, acts like a multifunctional protein which participates in neurotransmitter release at distinct steps. There are also multiple lines of evidence suggesting SNAP25 contributes directly to a spectrum of neuropsychiatric and neurological disorders^16^. In particular, Alzheimer's disease (AD), schizophrenia, attention deficient hyperactivity disorder, and epilepsy are included in this list^24,25^. Previous studies suggested SNAP25 as a biomarker in Alzheimer’s disease (AD), with reduced levels in AD^26^, as well as for Creutzfeldt-Jacob Disease patients^27^. Moreover, for Parkinson’s disease (PD) SNAP25 and other SNARE proteins have been associated with the pathogenesis of PD^26,28^. Therefore, the finding of a potential suppression of *SNAP25*, with or without ATF3 getting involved, in *SOD1*-mutant ALS patients might indeed be of importance and point to a general role in neurodegenerative diseases.

In this work, we also identified other genes with H3K27me3 mark around the TSS that are significantly suppressed in the *SOD1* mutant group. These genes are critical for neurodegenerative and neuroprotective studies in that they are mostly brain-specific genes that involve in neurogenesis and in the maintenance of a healthy neuronal network. Nuclear receptor subfamily 4 group A, or simply NR4A, proteins for instance are nuclear receptor (NR) transcription factors that play a regulatory role in neuronal development, inflammation, and memory formation^29^. The associated disorders are related to dopaminergic dysfunction, including PD, schizophrenia, and manic depression^30,31^. GADD45B, on the other hand, is a neuronal activity sensor induced by neuronal stress and essential for adult neurogenesis^32^. *KLF4*-encoded protein is expressed in neural stem cells, fine-tunes neurogenesis and axonal regeneration^33,34^, and has been associated with AD^35^. Interestingly, *SNAP25* is positively regulated by MAFB, one of the six genes mentioned above and involved in interneuron development^36^. SRY-box transcription factor 4, or SOX4, is one of the key elements that serves to promote neurogenesis and maintain neuronal properties in the course of vertebrate development^37^. Together, the key roles played by these genes in a healthy neuronal network implicate how vital it is to keep optimal levels of ATF3 expression in neurons.

## Conclusion

The current study presents evidence that extends the existing key role of ATF3 signaling in the pathogenesis of ALS caused by *SOD1* mutations. Two lines of evidence suggest that regulation of *SNAP25*, a target of ATF3, in ALS might be critical: firstly, *SNAP25* and *ATF3* seem to be epigenetically regulated by the repressive histone (H3K27me3) methylation, with consequent reduction of transcriptional activities. Secondly, ATF3 might modulate *SNAP25* expression directly by physically binding to *SNAP25* promoter region. These observations from the human blood linking impaired ATF3 signaling to *SOD1*-mutant ALS patients lay the groundwork for further investigation in brain sample of the same genotype for a more comprehensive understanding of the molecular mechanisms underlying ALS.

## Methods

### Study cohorts and ethical approval

Human blood sample collection was performed in accordance with the declaration of Helsinki and approved by the Ethic Committee of Ulm University. All volunteers gave informed written consent to participate in the study. ALS patients were diagnosed according to the El-Escorial criteria revised for ALS and considered as familiar ALS when two or more family members carried the same disease-causing mutation. Genotyping for a *SOD1* mutation was done by Sanger sequencing and testing for *C9orf72 hexanucleotide repeat expansion* by repeat primed PCR followed by Southern Blot confirmation. Healthy controls (HCs) without neurological conditions and confounding conditions affecting the immune system were chosen to match regarding age and sex to the patient cohort. From all participants, whole venous blood was collected in a standard Monovette™ blood drawing system (Starstedt) containing EDTA as anticoagulant. A summary of all clinical and demographic characteristics of the participants is provided in Supplementary Table S1.

### PBMC isolation

PBMCs of whole blood were isolated using Histopaque™-1077 density gradient centrifugation method. After washing the cells twice with DPBS, the PBMCs were subdivided for different approaches such as chromatin isolation and RNA extraction.

### ChIP-seq

Chromatin isolation from PBMCs was performed using the Chroma Flash Chromatin Isolation and Shearing Kit (Epigentek) according to manufacturer’s introduction. Briefly, PBMCs were cross-linked with 4% paraformaldehyde for 10 min at RT followed by quenching with 1.25 M Glycine. After washing, cells were lysed for 10 min on ice (buffer provided). The suspension was mixed and centrifugated at 3,000 × *g* for 5 min. The pellet was resuspended in extraction buffer (provided), incubated on ice for 10 min and regularly mixed. The lysate was sonicated in special sonication tubes (Diagenode) on high power settings for 3 runs of 30 min (30 s on/30 s off) using a Bioruptor Pico (Diagenode) to cut the DNA into 200 to 500 bp fragments. The samples were centrifuged at 13,800 ×* g* for 10 min at 4 °C and the supernatant was mixed with chromatin buffer (provided) in a 1:1 ratio. Sheared chromatin was pre-cleared with 1% BSA blocked protein A and G mag Sepharose Xtra magnetic beads (GE Healthcare) and subsequently incubated overnight at 4 °C on a rotator with antibody-labelled beads (10 µg antibody, H3K27m3 Merck or Chrom Pure Rabbit IgG from Jackson Immuno Research). Beads were washed with buffers of increasing stringency (low salt (0.1% SDS, 1% Triton X-100, 2 mM EDTA, 20 mM Tris–HCl [pH 8], 150 mM NaCl) to high salt (0.1% SDS, 1% Triton X-100, 2 mM EDTA, 500 mM NaCl)) and bound chromatin complexes were eluted (100 mM NaHCO_3_, 1% SDS). The DNA-antibody bead complex was reverse-crosslinked at 65 °C for 5 h (1300 rpm) and digested with proteinase K for further 2 h at 65 °C (1300 rpm) to remove proteins. Finally, DNA was purified using the ChIP DNA clean and concentrator kit (Zymo research). Libraries were generated using the NEBNext Ultra II DNA Library Prep Kit (Illumina) and subjected to single-end sequencing (101 bp) on a HiSeq2500 platform (Illumina).

### ChIP-seq data analysis

The ChIP-seq data set, generated using PBMCs from either healthy controls (n = 3) or ALS patients (n_*C9orf72*_ = 3 and n_*SOD1*_ = 2), was analyzed to produce genome-wide maps of H3K27me3 occupancy. For each sample, the adaptor contamination and overrepresented sequences identified after a round of pre-alignment quality control (FASTQC v0.11.9)^38^ were removed using Trim Galore! v0.6.7^39^. All sequencing libraries were confirmed for cross-species contamination that was likely missed at the QC step using FastQ Screen v0.15.2^40^. These refined single-end reads of length 101 bp were then aligned to the human genome (NCBI; GRCh38) using Bowtie v1.3.1^41^ one library at a time with the parameter "-m 1" to retain uniquely mapped reads only. As post-alignment QC steps, QualiMap v2.2.2^42^ and PreSeq v3.2.0^43^ were used to examine sequencing alignment for biases in the mapping and to estimate the complexity of the libraries, respectively. The R packages ChIPQC v1.20.0^44^ and PhantomPeakQualTools v1.2.2^45^ were used to confirm the quality of the aligned bam files based on the ChIP-seq guidelines by ENCODE consortium (e.g., FRiP > 1%, Relative CC ≈ 1, NRF ≈ 0.8, Qtag = {− 2, − 1, 0,1,2}). QC-confirmed bam files were subject to "callpeak" sub-command of MACS2 v 2.2.7.1^46^ with the following parameters: -t sample.sam -c input.sam -f SAM -g hs -B –nomodel –extsize 146 –broad –qvalue 0.01 –broad-cutoff 0.01. Considering the ENCODE guidelines, pairwise reproducibility (consistency between two biological replicates) for all samples in a sample group was calculated using IDR v2.0.4.2^47^ while the fraction of all mapped reads that overlap significant peaks was computed using the "featureCounts" command by SubRead v2.0.3^48^. A final decision was made for each individual library as to either include or exclude or pool replicates in a sample group after a thorough evaluation based on within- and between sample statistics as per the ENCODE metrics. ChIP-R v1.2.0^49^ was then used to combine through a rank-product test multiple experimental replicates from the same condition, and thereby, to generate a set of reproducible peaks per sample group for downstream analysis. To extract shared or unique peak regions by condition (i.e., *C9orf72*-specific, *SOD1*-specific, healthy control), the "intersect" command by bedtools v2.30.0^50^ was used. Post peak-calling steps, such as peak annotation and visualization, were performed using the R packages ChIPseeker v1.20.0^51^ and clusterProfiler v3.12.0^52^, respectively. Tests for over-representation within the scope of functional enrichment were performed using the R package ReactomePA v1.28.0^53^ or EnrichR v29.04.2022 at https://amp.pharm.mssm.edu/Enrichr/. An enrichment analysis was performed to identify target genes and colocalizing factors in the input data set based on the public ChIP-seq peak calls in the ChIP-Atlas dB^54^. STRING v11.5^55^ network analysis was used at a minimal interaction score of 0.400 (and at FET *p*-value = 1e-03). Visualization of the peaks across the genome was done using UCSC Genome Browser^56^ with custom tracks option enabled. The TF binding motif analysis and motif enrichments within peak regions were done using RSAT v2018^57^ and HOMER v4.11^58^ ("findMotifs.pl" command) with default parameters against the vertebrate and human backgrounds, respectively. Finally, cytoHubba v1.0.0^59^ was used to predict important nodes and to explore sub-networks in a given network by several topological algorithms.

### RNA-seq data analysis

The public (GSE106443 and GSE115259) RNA-seq data set^17^ generated using PBMCs from either healthy (n = 3) or *SOD1*-positive ALS (n = 3) subjects were prepared and analyzed separately. The "alternate" differential expression workflow, a modified version of the "new" Tuxedo pipeline at http://ccb.jhu.edu/software/stringtie/index.shtml?t=manual#deseq was used for the expression data analysis in this context. In brief, the data sets composed of paired-end reads of length 75 bp were first preprocessed to remove contaminating and overrepresented sequences and then rRNA contamination, and finally repaired to produce an interleaved fastq files using Trim Galore! v0.6.7^39^, HISAT2 v2.2.1^60^, and the "repair.sh" command of BBTools v37.62^61^, respectively. After two rounds of pre-alignment quality control (FASTQC v0.11.9)^38^ before and after preprocessing, the direction of the strandedness of all libraries have been confirmed using the "infer_experiment.py" script of the RSeQC v5.0.1^62^. The sample size of the control group was initially seven but dropped to three after low quality libraries were filtered out. Read alignment was performed using HISAT2 v2.2.1, default parameters with "min-intronlen 50" and “max-intronlen 2,000,000”, the human genome (NCBI; GRCh38) and the corresponding transcriptome annotation. As a post-alignment QC step, RSeQC v5.0.1 was used to confirm the mapping files. Transcript abundances were estimated for only known genes using StringTie v2.1.1^63^, generating one read coverage table per library. The output files obtained at this step were processed to identify differentially expressed (DE) transcripts using the R package Ballgown v2.16.0^64^ while the same files were first preprocessed using the author-supplied script “prepDE.py” at the website above for a proper format conversion before gene-level expression analysis. These preprocessed files were then fed into the R package DESeq2 v1.24.020^65^ for differential expression analysis at the gene level, whose results were used in the rest of the downstream analysis presented here. The "batch" covariate was included in this analysis to account for the technical variation associated with different batches. Differentially expressed genes (DEGs) were defined at an adjusted *p*-value (i.e., *q*-value; BH-corrected) cutoff < 5%, which is also applicable to the rest of the workflow. Genome-wide heatmaps and Venn diagrams were generated using the packages pheatmap v1.0.12 and gplots v3.0.1.1 in R^66^ respectively, taking the FPKM-normalized read counts as input. The same input used for the differential expression analysis above was analyzed using the GUI-based GSEA software v3.0^67^ to identify any gene sets in a large set of pre-defined biological processes (namely, the c2.cp.v7.5.1.symbols.gmt database) that are enriched between conditions. The number of permutations used to generate a reasonable null distribution in this context was chosen to 500 for stringent enrichment criteria (nominal *p*-value < 0.05, |NES|> 1.40). In silico validation of a selected pair of significant DEGs was done using receiver operating characteristics (ROC) curve with the area under curve (AUC) calculations by the R package pROC v1.18.0^68^, assessing discrimination ability of these genes for different conditions. Efficacy evaluation: AUC = 0.5 means non-efficiency, 0.5 < AUC < 0.7 means a modest level of efficiency, AUC > 0.7 means high efficiency.

### Cell Culture and Transfection

HEK293 cells were cultivated at 37 °C in 5% CO_2_ in DMEM (Life technologies) supplemented with 10% FBS (Sigma). Cells were transfected 24 h after plating using calcium phosphate co-precipitation as previously described^69^ with minor modifications. For the transfection master mix of a well from a 6-well plate, 4 µg DNA were supplemented with 200 µl H_2_O, and further completed with 24 µl 2.5 M CaCl_2_, 240 µl 2X HBS and 4 ml DMEM + 2% FBS. Afterwards, cells were cultivated at 37 °C in 5% CO_2_ for additional 24 h.

### Quantitative real-time PCR (qRT-PCR)

Total RNA was isolated from PBMCs using RNeasy Plus Mini Kit (Qiagen) with DNAse I digestion. 1 µg of total RNA was reverse-transcribed to cDNA using the iScript™ cDNA Synthesis Kit (Bio-Rad). Quantitative real-time PCRs were run on a CFX96 Real Time System (Bio-Rad) using iQ™ SYBR® Green Supermix (Bio-Rad). Oligonucleotides for qRT-PCRs are listed below. The relative mRNA expression level was analyzed by 2^-∆∆Ct^-method.

**Table:** 

Transcript	Direction	Sequence 5'–3'	Company
ATF3 occupancy on peak SNAP25	Forward	AGCCTGGCTCTGACGTAGTTC	Sigma
	Reverse	CTTGTGGCTCTCGTTGCTGTG	Sigma
H3K27me3 peak on SNAP25	Forward	ATGCAGTTGCGGGATGAAC	Sigma
	Reverse	ACCTGCGTGTTTTCACCTTC	Sigma
ATF3	Forward	GTGATTCAGCAGGCCCTTCCC	Sigma
	Reverse	AAGAATGGCCAGTGTGTTAAGGC	Sigma
B2M	Forward	AGATGAGTATGCCTGCCGTG	Sigma
	Reverse	GCGGCATCTTCAAACCTCCA	Sigma
Pol2R	Forward	TTGTGCAGGACACACTCACA	Sigma
	Reverse	CAGGAGGTTCATCACTTCACC	Sigma
RNU6	Forward	CTCGCTTCGGCAGCACAT	Sigma
	Reverse	AACGCTTCACGAATTTGCGT	Sigma
TBP	Forward	CCCATGACTCCCATGACC	Sigma
	Reverse	TTTACAACCAAGATTCACTGTGG	Sigma

### Western blotting

HEK cells were lysed in BEX buffer (20 mM NaCl, 0.6% sodium deoxycholate, 0.6% Igepal, 25 mM Tris, protease inhibitors). The samples were normalized to equal protein amounts by BCA assay (Thermo Fisher Scientific). 30 µg protein samples were separated on a 12% Tris–glycine gel by using a XCell Sure Lock™ Mini-Cell Electrophoresis system and transferred to a nitrocellulose membrane using a XCell Sure Lock™ Blot Module (Thermo Fisher Scientific). Following antibodies were used: rabbit-anti-ATF3 (1:500, #PA5-106898, Invitrogen), rabbit-anti-GAPDH (1:5000, 10494-1-AP, proteintech) and anti-rabbit IgG HRP conjugate as secondary antibody (1:10000, #W401B, Promega). Images were acquired in a FUSION SOLO S (Viber) with Luminata Forte HRP substrate (Merck).

### Statistics

A parametric F-test was used to detect differential expression (significant changes in mean gene expression) by Ballgown while a Wald test used by DESeq2. When broad peaks are called using MACS2, the no-model option is automatically set as well, as addressed elsewhere^46^. All functional enrichments were performed using Fisher’s Exact Test (FET) or an alternate hypergeometric test while motif enrichments were done using a hypergeometric test (in HOMER) or a binomial or a chi-squared test (in RSAT). A *p*-value or FDR < 0.05 denotes significance within the scope of this work. The expected distribution type of input data and soundness of underlying assumptions were confirmed prior to statistical analysis. Statistical analysis of relative expression levels was carried out with GraphPad Prism (v8.0). Statistical significance between two groups was tested with an unpaired, parametric t-test. *p*-values < 0.05 were considered as significant.

### Ethics approval and consent to participate

All human experiments were performed in accordance with the declaration of Helsinki and approved by the Ethics Committee of Ulm University. Informed consent was obtained from all participants included in the study.

## Supplementary Information

Supplementary Information.

## Data Availability

The samples in the ChIP-seq data set presented within the scope of this work have been deposited in the NCBI’s Gene Expression Omnibus (GEO) database under the accession GSE223834 in accordance with the MINSEQE standards^70^.

## Abbreviations

ATF: Activating transcription factor
AD: Alzheimer’s disease
ALS: Amyotrophic lateral sclerosis
AUC: Area under the curve
ChIP-seq: Chromatin immunoprecipitation following sequencing
*C9orf72*: *Chromosome 9 open reading frame 72*
CRE: Cis-regulatory element
DEG: Differentially expressed gene
FC: Fold change
GSEA: Gene set enrichment analysis
*GADD45B*: *Growth arrest and DNA damage inducible beta*
HC: Healthy control
*KLF4*: *Kruppel-like factor 4*
*MAFB*: *MAF BZIP Transcription factor B*
NES: Nuclear export signal
*NR4A*: *Nuclear receptor subfamily 4A*
PD: Parkinson disease
PBMCs: Peripheral blood mononuclear cells
SNARE: Soluble N-ethylmaleimide-sensitive-factor attachment receptor
*SOX4*: *SRY-Box Transcription Factor 4*
*SOD1*: *Superoxide dismutase 1*
*SNAP25*: *Synaptosome associated protein 25*
TF: Transcription factor
TSS: Transcription start site
H3K27me3: Trimethylation of lysine 27 on histone H3

## Code availability

The analysis pipeline and custom-made scripts used within the scope of this project are available from the corresponding author on reasonable request.
