# Supplementary material for: Impaired ATF3 signaling involves SNAP25 in SOD1 mutant ALS patients
Source: Sci Rep. 2023 Jul 25;13:12019. doi: 10.1038/s41598-023-38684-8 (PMC10368635; doi:10.1038/s41598-023-38684-8)
Supplement: Supplementary file 1 — Supplementary Information. [file 41598_2023_38684_MOESM1_ESM.pdf]

Impaired ATF3 Signaling Involves SNAP25 in SOD1 Mutant ALS Patients

Supplementary Files

Volkan Yazar[1, θ] , Julia K. Kühlwein[2, θ], Antje Knehr[2], Veselin Grozdanov[2], Arif B. Ekici[3], Albert C. Ludolph[1, 2], Karin M. Danzer[1,2,*]

[1] German Center for Neurodegenerative Diseases (DZNE), Ulm, Baden-Wuerttemberg, 89081, Germany

[2] Department of Neurology, University Clinic, University of Ulm, Ulm, Baden-Wuerttemberg, 89081, Germany

[3] Institute of Human Genetics, University Clinic Erlangen, Friedrich-Alexander-University Erlangen-Nürnberg, Erlangen, Bayern, 91054, Germany

[θ] These authors contributed equally.

*Correspondence:
Prof. Dr. Karin M. Danzer
karin.danzer@dzne.de
+49 731 500 63049
Albert-Einstein-Allee 11
89081 Ulm, Germany

Supplementary Figure S1.

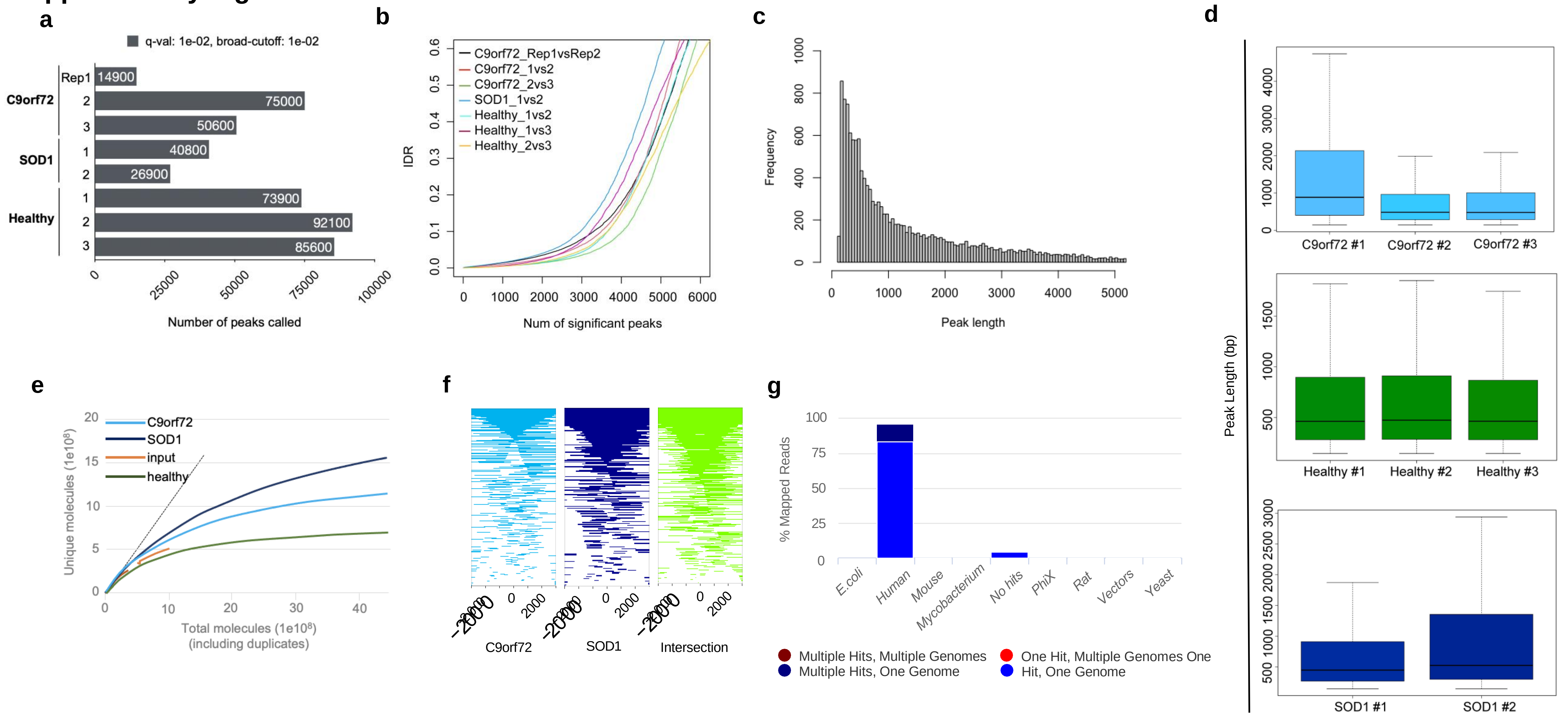

Supplementary Figure S1: Diagnostic plots for H3K27me3 peaks called in two different ALS and control samples in broad peak mode in accordance with the ENCODE guidelines. (a) Called at the FDR-corrected p-value cut-off 0.01 and the broad cut-off 0.01, the number of H3K27me3 peaks per biological replicate across the 3 sample groups (i.e., C9orf72, SOD1, and the healthy control). **(b)** The IDR-based pairwise reproducible analysis showing that a sufficient number of consensus peaks was identified for each sample group at an IDR of 0.05. **(c)** A histogram of peak lengths that follow a negatively skewed distribution, with an average length of 1766 bp. **(d)** Box-and-whisker plots for the peak lengths identified per replicate in different sample groups. **(e)** A library complexity analysis where the unique number of molecules are plotted as a function of the total number of molecules including duplicates per sample group. SOD1 and C9orf72 are particularly the most informative sample groups in the data set. **(f)** The genomic localization of the peaks that are either unique to either of the ALS sample groups or shared between both ALS groups excluding healthy controls, showing that the peaks are overlapping the gene TSS. **(g)** A bar plot confirming sequencing libraries for cross-species contamination.

Supplementary Figure S2.

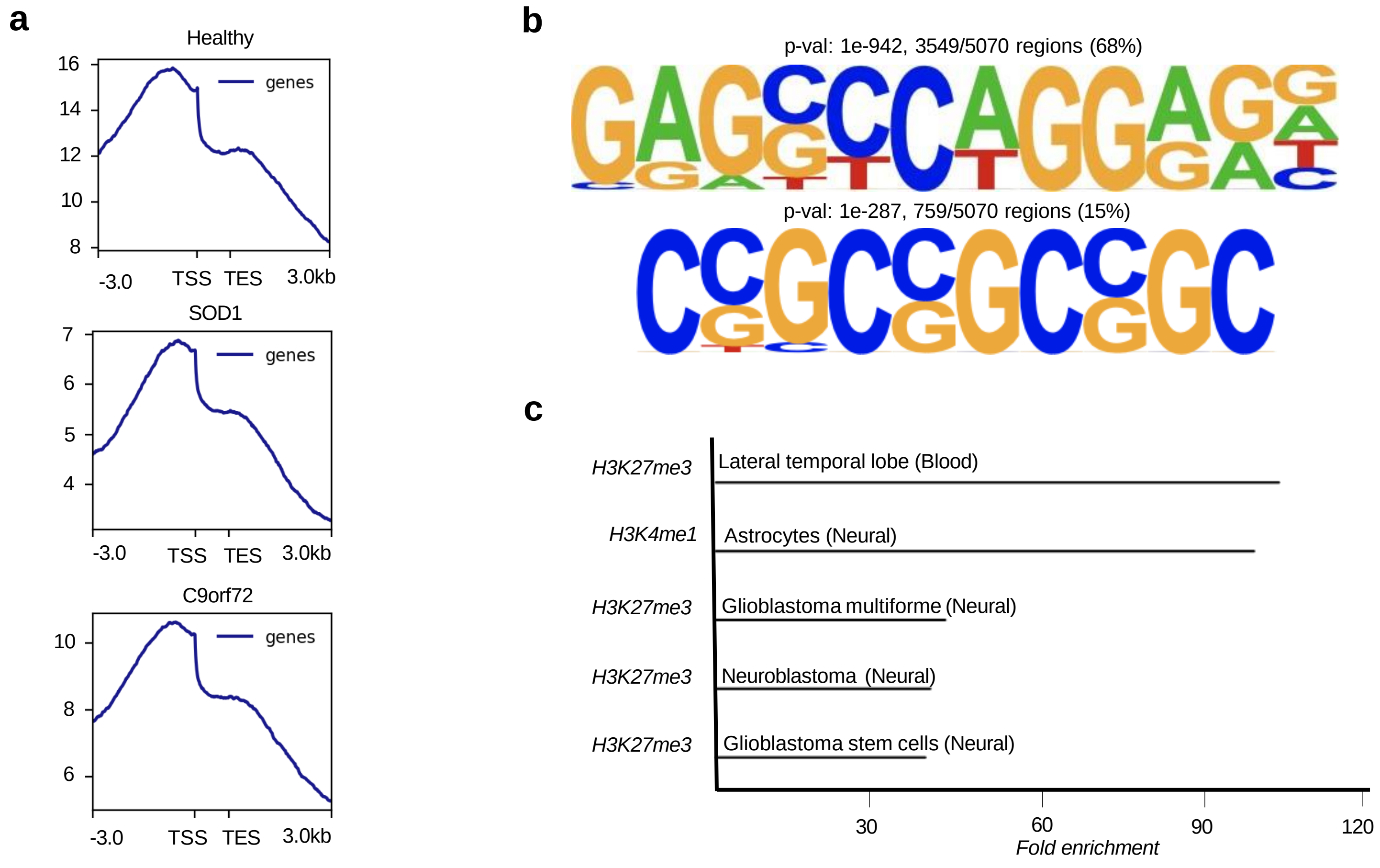

Supplementary Figure S2: Consensus sequence analyses and functional enrichment results of H3K27me3 peaks further confirm the methodologies used during library preparations and in data analysis. (a) The summary plots confirming the enrichment with the characteristic H3K27me3 signature around the promoter region (i.e., within +/-3 kb around the TSS) of the associated genes for all sample groups compared. **(b)** The QC results by two different motif discovery algorithms (i.e., HOMER v4.9.1-6 and RSAT v1.0.0) confirming the enrichment of H3K27me3 binding motif in the peak-associated sequences, with the significance, the frequency of occurrence, and the corresponding percentage of occurrence. H3K27me3 consensus sequence is highly similar to that of H3K4me3 (upper logo) and of H3K27me1 (lower logo). **(c)** The outcome of an enrichment analysis based on the public ChIP-seq peak calls in the ChIP-Atlas dB, which identifies target genes and colocalizing factors of a given transcription regulator. Our experimental design for H3K27me3 ChIP-seq experiment performed on hPBMCs has been confirmed for the choice of both tissue and histone modification within the scope of this work.

Supplementary Figure S3.

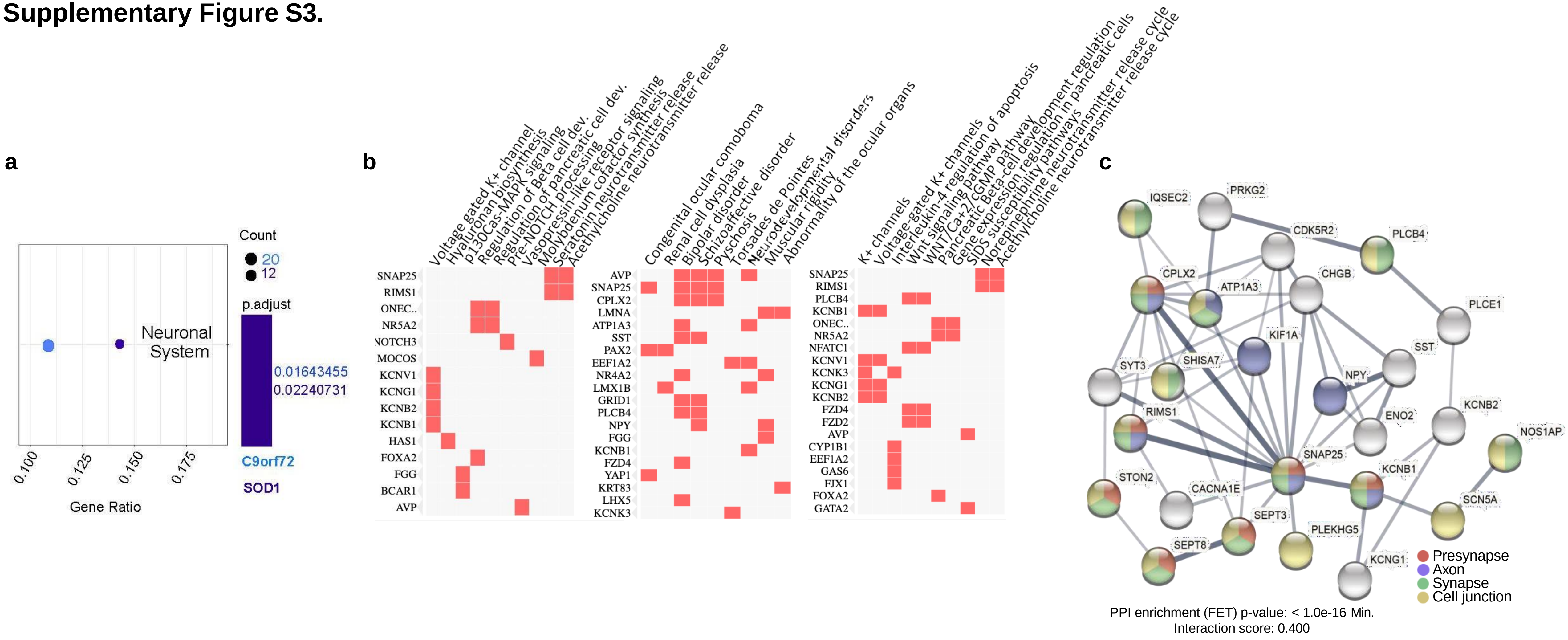

Supplementary Figure S3: SNAP25 is central to the interaction network composed of the peak-associated genes exclusively found in the SOD1-mutant patients. (a) The KEGG pathway analysis identified neuronal system as the most enriched ontology term in the peak-associated genes unique to either C9orf72 or SOD1 samples. This finding is also important in that H3K27me3 marks show, to some extent, similarity between blood and brain in fALS patients. **(b)** The most associated gene with the functional terms identified from the Reactome (2016: the leftmost plot), DisGenNet (the middle plot), and BioPlanet (2019: the rightmost plot) dBs enriched in the peak-associated genes is SNAP25. **(c)** A more comprehensive view of the interaction network predicted by STRING and mentioned in Fig. 2A. The number of nodes (genes): 26. The number of edges (interactions): 53. The average node degree: 1.83. The average local clustering coefficient: 0.292. The PPI enrichment p-value < 1.0E-16. The genes associated with the GO terms: “Presynapse” as red nodes, “Axon” as blue nodes, “Synapse” as green nodes, and “Cell junction” as yellow nodes. The edge thickness reflects the interaction strength between the corresponding nodes.

Supplementary Figure S4.

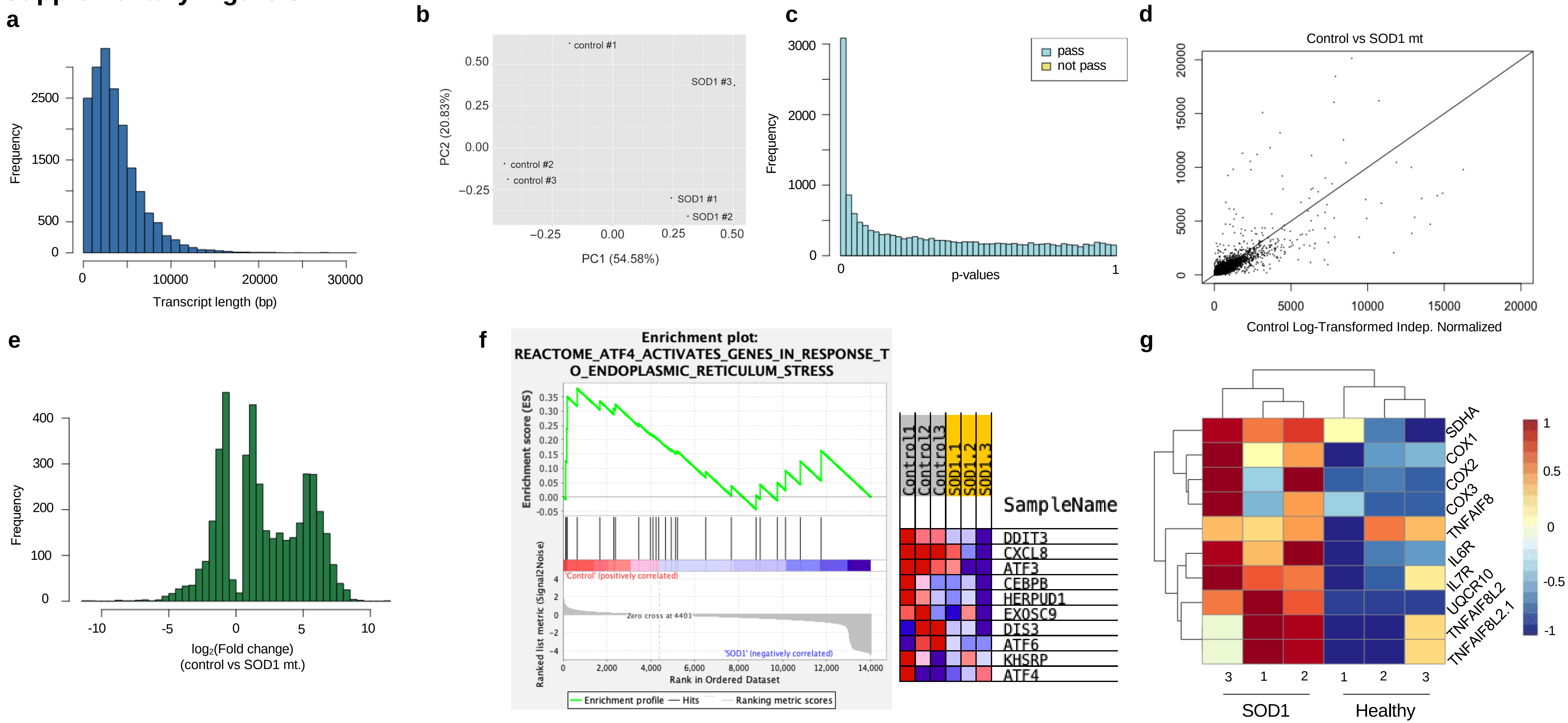

Supplementary Figure S4: ATF family proteins appear to act on the PBMC transcriptome in ALS caused by SOD1 mutations. (a) As a part of the bulk RNA-seq analysis pipeline, full length transcripts ($\tilde{x}$ = 1905 bp) have been first assembled. **(b)** A PCA plot showing the degree of separation between the sample groups along the PC1, which explains most (55%) of the variation in the data set. **(c)** A histogram inspecting the distribution of nominal p-values generated after differential expression analysis and removal of low count genes, which confirms the statistical approach used within the scope of this analysis. **(d)** A global scatter plot of normalized read counts per gene calculated using the DESeq2 differential expression protocol. Each dot denotes a gene that passed the filtering step. **(e)** A histogram showing the distribution of the log-transformed expression FC between conditions for the significant genes identified at an FDR-corrected p-value cut-off of 0.05. **(f)** A gene set enrichment analysis (GSEA) reported ATF family (ATF3/4) TF signaling as one of the most enriched gene sets in the expression data. The adjacent heatmap represents the expression profiles per replicate of the genes associated with that gene set, implying that SOD1 G93A mutant patient contributes mostly to these results. **(g)** Given a set of known molecular changes commonly observed in ALS blood, an attempt to rule out the possibility of a systemic issue, revealed a general coherency for the genes tested in this sample group.

Supplementary Figure S5.

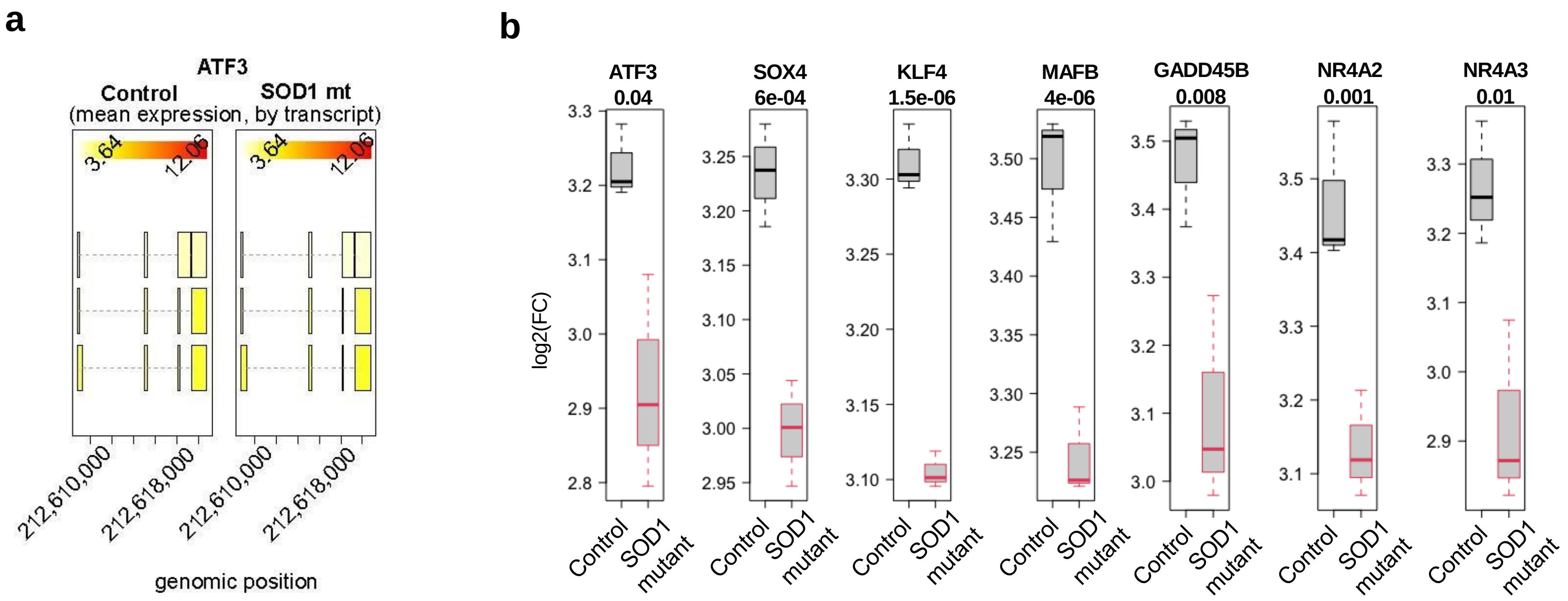

Supplementary Figure S5. ATF3 and the 1st shell interactors of ATF3 appear significantly downregulated in ALS PBMCs with SOD1 mutation. (a) A transcript-level differential analysis found no apparent change in ATF3 expression, unlike gene-level expression analysis. Horizontally stacked boxes (exons) linked with dashed lines (introns) in each line denote a transcript. **(b)** Differences in expression levels of ATF3 and of the 1st shell interactors of ATF3 between conditions. FDR-corrected p-values computed for each gene are also given underneath the corresponding gene symbols.

Supplementary Figure S6.

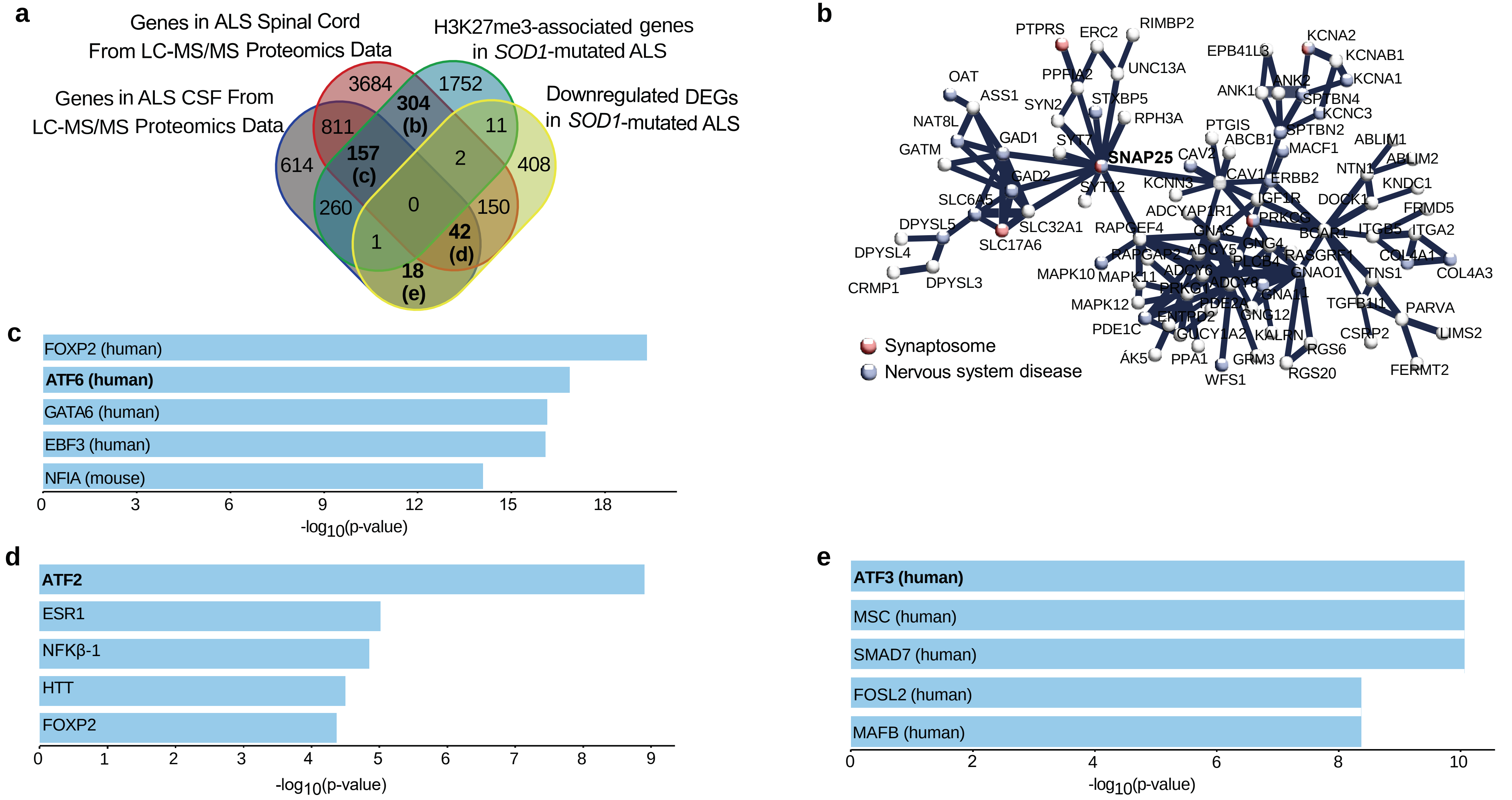

Supplementary Figure S6. ATF family TFs and SNAP25 ontology terms were found enriched in the shared genes when a publicly available proteomics data set generated from different CNS tissues of sporadic ALS patients was included in this study. (a) A 4-group Venn diagram was plotted to identify the shared genes associated with ALS in multiple data sets from different tissues independent of the mode of disease inheritance. The numbers in bold: the query sample sizes used for functional enrichment analysis. The letters in parenthesis: the panel labels with the corresponding enrichment analysis results. **(b)** SNAP25 was identified as a hub in the STRING interaction network of the 304 genes in (a). The PPI enrichment (FET) p-value < 1e-16. Minimum interaction score: 0.700 (high confidence). **(c-e)** ATF family TFs in such annotation libraries as the TF Perturbations Followed by Expression, the TF PPI, and the ARCHS4 TF Coexpression were shown to be the most likely common regulators of the 157, 42, and 18 target genes in (a), respectively.

Supplementary Table S1.

	fALS_*C9orf72*	fALS_*SOD1*	HC
**Number of samples**	5	4	6
**Number of replicates**	3	2	3
**Gender (f/m)**	1/4	3/1	3/3
**Age**	57.60 ± 13.67	55.00 ± 8.12	59.33 ± 12.09
**Age of Onset**	56.20 ± 13.08	48.75 ± 12.69	N/A
**bulbar/limb onset**	2/3	0/4	N/A
**ALSFRS**	41.80 ± 4.21	37.00 ± 9.56	N/A

Sample ID	Gender	Age	Mutation	Age of onset	side of onset (bulbar/limb)	ALSFRS	Replicate no.
fALS_1	male	60	*C9orf72 (1450-2600 repeats)*	59	limb	35/48	1
fALS_2	male	38	*C9orf72 (80-150 repeats)*	36	bulbar	46/48	2 (pooled)
fALS_3	male	54	*C9orf72 (1600-2300 repeats)*	54	limb	43/48	
fALS_4	male	60	*C9orf72 (1600-2450 repeats)*	60	limb	44/48	3 (pooled)
fALS_5	female	76	*C9orf72 (55-80 repeats)*	72	bulbar	41/48	
fALS_6	female	50	*SOD1 (V149G)*	50	limb	39/48	1 (pooled)
fALS_7	female	67	*SOD1 (I114T)*	64	limb	23/48	
fALS_8	male	53	*SOD1 (E101K)*	48	limb	44/48	2 (pooled)
fALS_9	female	50	*SOD1 (I104F)*	33	limb	42/48	
HC_1	male	70	-	-	-	-	1 (pooled)
HC_2	female	66	-	-	-	-	
HC_3	female	61	-	-	-	-	2 (pooled)
HC_4	male	38	-	-	-	-	
HC_5	female	68	-	-	-	-	3 (pooled)
HC_6	male	53	-	-	-	-	

Supplementary Table S1. Clinical characteristics of the study participants. The upper table displays a summary of clinical information from volunteers participated in the ChIP-seq study while the lower table lists each proband individually. Patients were considered as familiar ALS based on genetic inheritance of the same ALS-causative mutation in at least two family members. Individuals with confounding conditions affecting the immune system were excluded. Genotyping for SOD1 was performed with Sanger sequencing and C9orf72 was tested with repeat primed PCR following Southern blot. Healthy controls (Ctrl) and ALS patients were matched for age and gender. Data are given as mean ± SD.

Supplementary Table S2.

Sample Group	Replicate no.	Fragment Length	Relative CC	NSC	Qtag	NRF	FRiP (%)	% of peaks at IDR < 0.05	No. of consensus peaks (q-val < 0.01, broad-cutoff = 0.01)	No. of consensus peaks (associated genes) unique to a group	No. of consensus peaks (associated genes) at the intersection excl. HC
fALS_ C9orf72	1	202	1.00	1.01	1	0.84	5.4	37.6, 37.6, -	5070	545 (414)	380 (297)
	2	205	1.05	1.03	1	0.84	16.1	-, 16.1			
	3	208	1.10	1.02	0	0.83	10.4	-			
fALS_ SOD1	1	201	0.96	1.01	1	0.83	6.9	23.1, -, -	3390	240 (195)	
	2	205	0.79	1.02	0	0.83	6.3	-, -			
**HC**	1	205	1.60	1.01	1	0.84	12.9	11.3, 10.7, -	6766	2462	-
	2	202	1.25	1.02	0	0.84	15.1	-, 10.3			
	3	201	1.20	1.01	0	0.84	13.0	-			

Supplementary Table S2. The quality control (QC) metrics for the H3K27me3 ChIP-seq data analysis. HC: healthy control. CC: cross-correlation coefficient. NSC: normalized strand cross- correlation coefficient. Qtag: quality tag (-2: very low, -1: low, 0: medium, 1: high, 2: very high). NRF: non-redundant fraction. FRiP: fraction of reads under peaks. IDR: irreproducible discovery rate.
